# Preconditioning of MSCs for Acute Neurological Conditions: From Cellular to Functional Impact—A Systematic Review

**DOI:** 10.3390/cells13100845

**Published:** 2024-05-16

**Authors:** Inês Serrenho, Susana Alves Ferreira, Graça Baltazar

**Affiliations:** 1 Centro de Investigação em Ciências da Saúde (CICS-UBI), Universidade da Beira Interior, 6200-506 Covilhã, Portugal; inesserrenho@fcsaude.ubi.pt (I.S.); susana.alves.ferreira@fcsaude.ubi.pt (S.A.F.); 2 Faculdade de Ciências da Saúde, Universidade da Beira Interior, 6200-506 Covilhã, Portugal

**Keywords:** mesenchymal stem cells, stem cell therapy, ischemic diseases, traumatic diseases, preconditioning, therapeutic potential

## Abstract

This systematic review aims to gather evidence on the mechanisms triggered by diverse preconditioning strategies for mesenchymal stem cells (MSCs) and their impact on their potential to treat ischemic and traumatic injuries affecting the nervous system. The 52 studies included in this review report nine different types of preconditioning, namely, manipulation of oxygen pressure, exposure to chemical substances, lesion mediators or inflammatory factors, usage of ultrasound, magnetic fields or biomechanical forces, and culture in scaffolds or 3D cultures. All these preconditioning strategies were reported to interfere with cellular pathways that influence MSCs’ survival and migration, alter MSCs’ phenotype, and modulate the secretome and proteome of these cells, among others. The effects on MSCs’ phenotype and characteristics influenced MSCs’ performance in models of injury, namely by increasing the homing and integration of the cells in the lesioned area and inducing the secretion of growth factors and cytokines. The administration of preconditioned MSCs promoted tissue regeneration, reduced neuroinflammation, and increased angiogenesis and myelinization in rodent models of stroke, traumatic brain injury, and spinal cord injury. These effects were also translated into improved cognitive and motor functions, suggesting an increased therapeutic potential of MSCs after preconditioning. Importantly, none of the studies reported adverse effects or less therapeutic potential with these strategies. Overall, we can conclude that all the preconditioning strategies included in this review can stimulate pathways that relate to the therapeutic effects of MSCs. Thus, it would be interesting to explore whether combining different preconditioning strategies can further boost the reparative effects of MSCs, solving some limitations of MSCs’ therapy, namely donor-associated variability.

## 1. Introduction

Mesenchymal stem cells (MSCs) are cells with the ability to self-renew and differentiate into mesoderm cells of the bone, cartilage, and adipose tissue (AD) [1]. These cells can be obtained from different sources, such as the umbilical cord (UC), placenta, AD, and bone marrow (BM), which are the most used [2].

MSCs have been used in the treatment of various diseases because of their low risk of triggering immune rejection [3], immunomodulatory, pro-angiogenic, anti-apoptotic, and antioxidative potential [1]. Moreover, the use of MSCs to treat neurological diseases has attracted great interest in recent years due to their potential to repair and regenerate damaged neural networks [4,5,6].

Currently, several studies are exploring if the modulation of culture conditions exposure to compounds or biomechanical forces, among others, influences the therapeutic potential of MSCs. Reports show that these stimuli can alter the proteome and secretome of MSCs [7,8], improve cell survival, increase differentiation efficiency, enhance paracrine properties, and improve migration and homing of the cells to the lesion site [9]. The modulation of stem cells’ proteome, and consequently their secretome, via exposure to manipulated microenvironments has emerged as a way of boosting their therapeutic potential. In this systematic review, preconditioning was considered when the cell microenvironment was manipulated with the aim of altering its proteome and secretome.

Although there is no extensive published work on the effects of various types of preconditioning on MSCs, the evidence that preconditioning positively modulates these cells has been growing. Therefore, this systematic review aims to gather information on the modulatory effects of various types of preconditioning, both at a cellular and functional level and identify the effects of each preconditioning strategy in different preclinical models of neurological conditions that arise from ischemic or traumatic events. This review only includes articles that study acute-onset neurological conditions because MSC treatment in these pathologies may potentially be more successful. In the case of neurodegenerative diseases, there is continuous neurodegeneration, unlike acute-onset pathologies, where an insult leads to the death of neural cells, but there is no progressive degeneration. Thus, if the process leading to the disease and the triggered mechanisms are mitigated, the injury process will not progress/worsen.

Preconditioning methods were organized into three main groups: use of extrinsic factors, differential oxygen pressure, and use of culture scaffolds or biomechanical forces.

## 2. Methods

### 2.1. Literature Search

**Figure 1:**
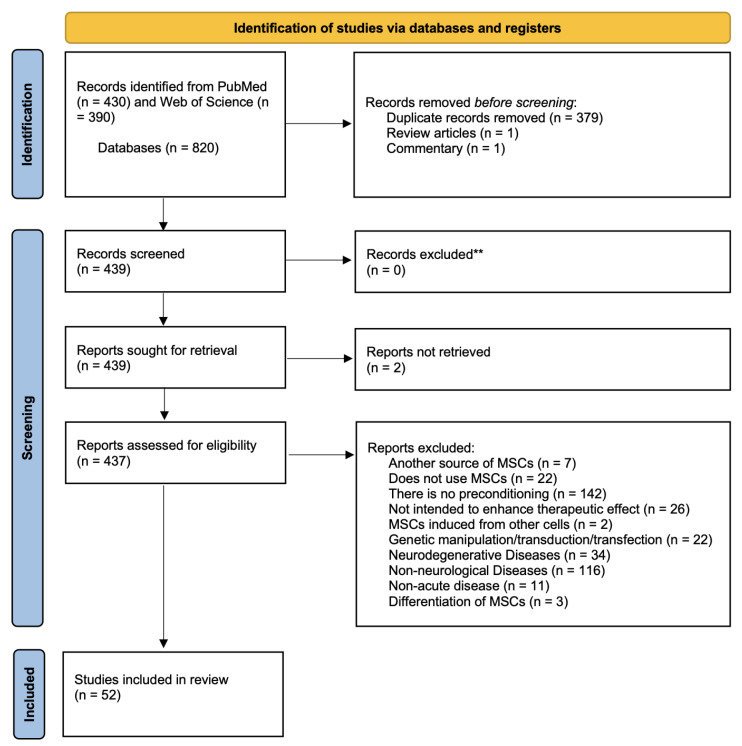
Flow diagram representing the literature search and exclusion criteria applied to select the studies included in this systematic review. MSCs: Mesenchymal stem cells.

The study was conducted according to the Preferred Reporting Items for Systematic Reviews and Meta-analyses guidelines (Supplementary File S1). It is registered in the OSF platform under n°: 10.17605/OSF.IO/BRSCU. The literature search was performed using two different databases (PubMed and Web of Science) on 4 May 2023. The literature search included the following search terms: (((pretreatment) OR (preconditioning) OR (preconditioned) OR (priming) OR (preconditioning) OR (conditioning) OR (conditioned) OR (primed)) AND ((Stroke) OR (Ischemia) OR (MCAO) OR (OGD) OR (Photothrombosis) OR (“Thromboembolic clot”) OR (Endothelin-1) OR (FeCl3) OR (“neurological disease”) OR (“neurological disorder”) OR (“brain injury”) OR (“brain trauma”) OR (neuro) OR (brain) OR (“spinal cord injury”) OR (“traumatic brain injury”) OR (“hypoxic-ischemic encephalopathy”)) AND ((“mesenchymal stem cells”) OR (“mesenchymal stromal cells”) OR (MSC) OR (MSCs)) NOT (review) AND ((“umbilical cord”) OR (“bone-marrow”) OR (“bone marrow”) OR (adipose)) NOT ((cardio*) or (kidney))). Not all the above terms exist as MESH terms, so the search was carried out using only keywords. Only peer-reviewed articles published in English were selected. Duplicates were manually removed from the search results. Two authors conducted the literature search and independently screened the abstracts and the full text of the studies. Any disagreements were settled by a discussion with the third author. The literature search and exclusion criteria applied to select the studies are shown in Figure 1.

### 2.2. Inclusion and Exclusion Criteria 

The literature search and exclusion criteria used are summarized in Figure 1. Considering our review question and following a PICO structure, we only included preclinical studies in cell cultures or animals that always included a control group, evaluating the effects of various preconditioning protocols on MSCs. In the results, we included effects at a cellular level, as well as functional effects at a motor and cognitive level. All review articles and commentaries were excluded. We also excluded studies that did not use MSCs or that did not pre-condition them, those that did not aim to increase the therapeutic effect, those that used MSCs that had been induced from other cells, genetic manipulation, transduction or transfection protocols, studies in neurodegenerative, non-neurological or chronic diseases or MSCs differentiation protocols. Two articles were excluded because the full text was not available.

### 2.3. Data Extraction 

The following information was extracted from the included articles: type of preconditioning, the pathology model, the type of experimental model (in vivo, in vitro, or both), the species, the source of MSCs, the administration time of the cells and its dose, and the main results. This information is summarized in Table 1, Table 2 and Table 3. The organization of the extracted data were performed considering the type of injury (ischemic or traumatic) and within each of these sections the data were organized according to the type of preconditioning. The literature search led to the identification of 820 articles, which were analyzed. From these only 439 were considered eligible, and after a more thorough evaluation only 52 fitted the defined criteria. None of the studies include information regarding randomization and blinding of the experimental design, so the risk of bias has not been assessed. The data and information recovered from each included study were categorized into three different categories: (1) Evaluate the effect of preconditioning on MSCs alone; (2) Evaluate the effectiveness of preconditioning in ischemic brain injuries; (3) Evaluate the effectiveness of preconditioning in spinal cord injury (SCI) and traumatic brain injury (TBI). These categories correspond to Section 3.1, Section 3.2 and Section 3.3 of this review, respectively.

## 3. Results and Discussion

### 3.1. Effect of the Preconditioning Method on the MSCs Phenotype and Characteristics

**Figure 2:**
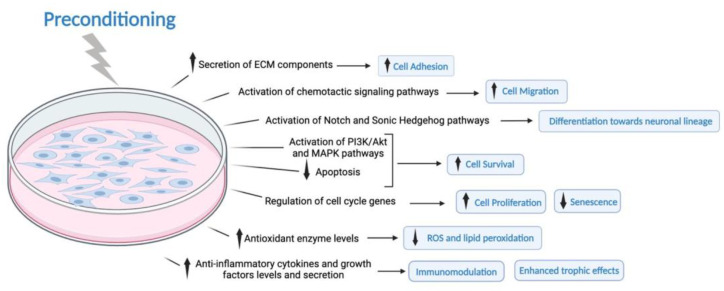
The network of pathways and mechanisms triggered by preconditioning MSCs. The release of ECM orchestrates cell adhesion and migration. Preconditioning of MSCs activates, to a higher extent, chemotactic pathways that translate into increased cell migration. The activation of Notch and Sonic Hedgehog pathways stimulates the MSCs’ differentiation towards neuronal lineage. The modulation of cell cycle genes upon preconditioning mitigates senescence and increases MSC proliferation. Moreover, several preconditioning techniques increased the expression of antioxidant enzymes, which collectively contribute to decreased ROS and lipid peroxidation. This, in addition to the activation of cell survival pathways and decreased apoptosis, increases the survival of MSCs upon exposure to adverse stimuli. Concurrently, up-regulated anti-inflammatory cytokines and growth factors contribute to the immunomodulation of the cells and enhanced trophic effects. These mechanisms underscore the multifaceted nature of preconditioning-induced modulation that translates into enhanced MSCs’ therapeutic potential for injuries to the central nervous system. Abbreviations: ECM—extracellular matrix; MAPK—mitogen-activated protein kinase; PI3K/Akt—phosphoinositide 3-kinase/protein kinase B pathway; ROS—reactive oxygen species; ↑—upregulation; ↓—downregulation.

This review includes thirty-seven original articles that evaluated the impact of preconditioning methods on the nature of MSCs, namely their phenotype, and characteristics. Twenty-one correspond to in vitro studies and sixteen were developed both in vitro and in vivo. Within these thirty-seven articles, eight different types of preconditioning were used, namely biomechanical forces, chemical substances, culture scaffolds, manipulation of cell culture supplementation, different oxygen pressure, exposure to lesion mediators, inflammatory factors, and ultrasound and magnetic fields. The relevant information for each of the articles is summarized in Table 1 and Figure 2. To ease the collection of information, these types of preconditioning were organized into three subgroups: extrinsic factors, different oxygen pressure, and culture scaffolds and biomechanical forces.

**Table 1:** Studies focused on the effect of the preconditioning method on the MSCs’ phenotype and characteristics.

Ref.	Type of Preconditioning	Preconditioning	Pathology	Type of Study	Source	Administration Time (dpi)	Duration (h)	Dose Quantity Intensity
[10]	Biomechanical Forces	Microfluidic devices	Traumatic Brain Injury	in vitro and in vivo	Human BM	1 or 3	3, 6 or 8	15 dyne/cm^2^
[11]	Chemical substances	Curcumin	Ischemia and reperfusion	in vitro	Rat BM	-	2	1, 5, 10 or 20 μM
[12]	Chemical substances	Astaxanthin	Ischemic stroke	in vitro	Human AD	-	?	2–128 μM
[13]	Chemical substances	Calpain inhibitor (MDL28170) and hypoxia cm or tunicamycin	Spinal Cord Injury	in vitro and in vivo	Rat BM	7	1 MDL28170 and 24 hypoxia cm or tunicamycin	1–10 µM MDL28170 and hypoxia cm (0%) or 0, 1, 3, 10 mg/mL tunicamycin
[14]	Chemical substances	Roxadustat	Ischemic stroke	in vitro and in vivo	Rat BM	1	24	10 μmol/L
[15]	Chemical substances	Fasudil	Spinal Cord Injury	in vitro and in vivo	Rat BM	7	12, 24, 36, 48, and 72	3, 10, 30, and 100 μmol/L
[16]	Chemical substances	Isoflurane	Ischemic stroke	in vitro and in vivo	Rat BM	-	2, 4, 6, 12, and 24	1–10%
[17]	Chemical substances	Artemisinin	Ischemic stroke	in vitro	Rat BM	-	24	0.1–100 μM
[18]	Chemical substances	Sevoflurane	Ischemia and reperfusion	in vitro	Rat BM	-	2	2%
[19]	Chemical substances	Hydrogen sulfide	Ischemic stroke	in vitro and in vivo	Rat BM	1	5, 15, 30, 60, 120, and 240 min	0.1, 0.5, 1, 5, 10 and 50 μM in vitro and 1 μM in vivo
[20]	Chemical substances	Modulation of autophagy with rapamycin and 3-MA	N/A	in vitro	Human AD	-	1, 4, 12, 24, and 48	Rapa 500 nM or 3-MA 5 mM
[21]	Chemical substances	Rosmarinic acid	Ischemic stroke	in vitro	Rat AD	-	4 or 24	0.2–6 μM
[22]	Chemical substances	Lycopene and hypoxia	Ischemic stroke	in vitro	Mouse BM	-	1 with lycopene and 6 of hypoxia with lycopene	1 h with lycopene (0, 1, 2, 5, 10, 20, 50 µM) and 6 h of lycopene with/without 20 µM LY294002 (PI3K/Akt inhibitors)
[23]	Culture scaffolds/3D culture	Graphene Oxide-Substrate	Peripheral nerve injury	in vitro	Human AD	-	72	-
[24]	Culture scaffolds/3D culture	Encapsulated in 3D hydrogels derived from human fibrin or platelet lysate	N/A	in vitro	Human Wharton’s Jelly	-	Duration of the culture	-
[25]	Different culture supplementation	Platelet lysate and G-CSF	Ischemic stroke	in vitro and in vivo	Human BM	7	?	5% HPL + 0.1 μM G-CSF
[26]	Different culture supplementation	Growth medium with neuregulin1-beta1, bFGF, PDGF-AA and forskolin	Peripheral nerve injury	in vitro and in vivo	Human AD	0	2 weeks	200 ng/mL neuregulin1-beta1, 10 ng/mL bFGF, 5 ng/mL PDGF-AA, and 14 mM forskolin
[27]	Different culture supplementation	bFGF, B27 and kanamycin	N/A	in vitro	Human AD and UC	-	7 days	0.1 to mg/mL
[9]	Different oxygen pressure	Hypoxia	Ischemic stroke	in vitro and in vivo	Rat BM	1	0, 4, 8, 12, and 24	1% O_2_
[28]	Different oxygen pressure	Hypoxia	Ischemia and reperfusion	in vitro	Rat BM	-	24	1% O_2_
[29]	Different oxygen pressure	Hypoxia	Ischemic stroke	in vitro	Human BM	0	24	1% O_2_
[30]	Different oxygen pressure	Hypoxia	Ischemic stroke	in vitro	Mouse BM	-	24	0.5% O_2_
[31]	Different oxygen pressure	Hypoxia	Ischemic stroke	in vitro and in vivo	Rat BM	1	24, 48, 72	0.5% O_2_
[32]	Different oxygen pressure	Hypoxia	Spinal Cord Injury	in vitro and in vivo	Rat BM	0	48 or 72	1% O_2_
[24]	Different oxygen pressure	Physioxia	N/A	in vitro	Human Wharton’s Jelly	-	Duration of the culture	5% O_2_
[33]	Different oxygen pressure	Hypoxia	N/A	in vitro	Human UC	-	24	1% O_2_
[34]	Different oxygen pressure	Hypoxia	N/A	in vitro	Canine BM	-	6, 12, and 24	1% O_2_
[35]	Exposure to lesion mediators	Cerebral tissue extracts from TBI rats	Traumatic Brain Injury	in vitro	Human BM	-	-	20% TBI tissue extract supernatant
[36]	Exposure to lesion mediators	Stroke patient serum	Ischemic stroke	in vitro and in vivo	Human BM	1	-	10%
[37]	Exposure to lesion mediators	SCI patient plasma	Spinal Cord Injury	in vitro and in vivo	Human BM	0 and once a week for 8 weeks	Duration of the culture	10%
[38]	Exposure to lesion mediators	Activated microglia	Ischemic stroke	in vitro	Rat BM	-	24	?
[39]	Inflammatory factors	IL-1α, IL-1β, TNF-α or IFN-γ	N/A	in vitro	Human BM	-	24	1, 10, 50 or 100 ng/mL
[40]	Inflammatory factors	Recombinant human IFN-γ	Periventricular leukomalacia	in vitro and in vivo	Human UC	0	24	10 ng/mL
[41]	Ultrasound and magnetic fields	Electromagnetic field	N/A	in vitro	Human BM	-	24, 72, 120	60 Hz
[42]	Ultrasound and magnetic fields	Low intensity pulsed ultrasound	Spinal Cord Injury	in vitro and in vivo	Rat BM	7	72	10, 30, 50, 70 mW/cm^2^, 3 min/d
[43]	Ultrasound and magnetic fields	Low frequency pulsed electromagnetic field	Crush-injured nerve	in vitro and in vivo	Rat BM	0	1	50 Hz, 1 mT

AD—adipose tissue; BM—bone marrow; cm—centimeter; dpi—days post injury; FGF—fibroblast growth factor; G-CSF—granulocyte-colony stimulating factor; h—hours; HPL—human platelet lysate; Hz—hertz; IFN—interferon; IL—interleukine; L—liter; min—minute; mT—militesla; N/A—not applicable; SCI—spinal cord injury; TBI—traumatic brain injury; TNF—tumor necrosis factor; UC—umbilical cord; ?—not reported.

#### 3.1.1. Extrinsic Factors

##### Chemical Substances

Twelve studies explored the impact of preconditioning MSCs derived from adipose tissue or bone marrow with chemical substances. The various chemical agents used in these studies share the ability to promote increased survival of MSCs, neuroprotection, and increased MSC migration.

Ten studies reported an increase in MSC survival after exposure to the preconditioning substances. Incubation of MSCs with the antioxidant lycopene protected the cells from serum deprivation- and hypoxia-induced apoptosis reduced the production of reactive oxygen species (ROS) and decreased the expression of inducible nitric oxide synthetase [22]. MSCs preconditioned with a hydrogen sulfide donor presented less hypoxia-induced apoptosis, which was attributed to the activation of cell survival pathways [19].

The use of phytochemicals has also been studied as a form of promoting MSC survival. Incubation of glucose- and serum-deprived AD-MSCs with rosmarinic acid, a polyphenolic phytochemical, promoted AD-MSCs survival by decreasing the levels of ROS, lipid peroxidation, the percentage of cells in the sub-G1 stage, and necrosis [21]. Additionally, the use of curcumin as a preconditioning strategy for BM-MSCs reduced cell viability loss, mitochondrial dysfunction, ROS accumulation, cell nuclei condensation, lactate dehydrogenase (LDH) leakage [11]. This strategy also increased caspase-3 activity after hypoxic insults via destabilization of hypoxia-inducible factor (HIF)-1α and activation of proteins related to cell survival pathways [11]. Moreover, pretreatment with artemisinin, a sesquiterpene endoperoxide widely used in Chinese traditional medicine, improved BM-MSCs survival after exposure to hydrogen peroxide [17]. The anti-apoptotic effect was accompanied by reduction of ROS production, increase in antioxidant enzyme activities, decreased caspase-3 activation, LDH release, and increased Erk1/2 phosphorylation. Remarkably, when the Erk1/2 pathway was inhibited, artemisinin protection was abrogated, suggesting that this pathway was involved in these protective effects [17]. Astaxanthin was reported to protect AD-MSCs from cell apoptosis and oxidative stress induced by hydrogen peroxide [12]. This xanthophyll carotenoid also increased the expression of Nuclear Erythroid 2-Related Factor 2 (Nrf2), a key transcription factor of antioxidant enzymes [12]. These studies reveal the potential of using phytochemicals as a strategy of preconditioning, due to their ability to enhance MSCs survival and protect these cells from various stressors, such as oxidative stress and hypoxic insults.

Another strategy being studied to improve MSC survival rate is the modulation of HIF-1α levels and activation of the HIF-1α-related pathways. Indeed, preconditioning with Roxadustat, a drug used for treating anemia in chronic kidney disease patients, increased the viability and proliferation of BM-MSCs under oxygen and glucose deprivation (OGD) [14]. The authors reported that Roxadustat decreased BM-MSCs apoptosis rate after OGD [14]. AD-MSCs pretreated with Rapamycin, an autophagy inducer, preserved their stemness and up-regulated inflammatory mediators known to promote tissue repair and regeneration [20]. Because the exposure to 3-Methyladenine (3-MA), an autophagy inhibitor, resulted in similar effects, it is probable that autophagy is not the mechanism behind the reported effects. Nonetheless, only Rapamycin-treated AD-MSCs had increased tumor necrosis factor (TNF)-α-stimulated gene-6 and IL-1β, indicating additional enhancement of immunomodulatory potential [20].

Inhaled anesthetics have also been used to precondition MSCs due to their ability to induce tolerance against ischemic injury [44]. Preconditioning of MSCs with sevoflurane [18] or isoflurane [16] reduced apoptosis and promoted in vitro survival of MSCs. However, whereas short exposures to low isoflurane concentrations promoted in vitro survival and migration of BM-MSCs, long exposures and high concentrations had the opposite effect. Moreover, the authors reported that the preconditioning with isoflurane up-regulated the expression of HIF-1α, stromal-derived factor-1 receptors, and activation of Akt in BM-MSCs cultures [16], both associated with cell survival. Exposure to sevoflurane increased the migration of rat BM-MSCs and the expression of HIF-1α, HIF-2α, vascular endothelial growth factor (VEGF), and p-Akt/Akt, which were reduced by hypoxia preconditioning (HP) and serum deprivation in vitro. Additionally, the co-culture of sevoflurane preconditioned BM-MSCs with neuron-like PC12 cells subjected to ischemia reduced neuronal cell apoptosis, supporting the neuroprotective effect induced by this strategy [18].

In addition to the effects reported so far, preconditioning with Fasudil, a potent Rho kinase inhibitor, significantly enhanced BM-MSCs migration ability and increased the formation of actin stress fibers [15].

##### Inflammatory Factors

Wise and colleagues (2022) combined two types of preconditioning: chemical substances that regulate autophagy and inflammatory cytokine interferon-gamma (IFN-γ). Exposure of MSCs-pretreated with Rapamycin (autophagy inducer) to IFN-γ increased inflammatory cytokines and TGF-β levels. Curiously, this effect was not observed in cells pretreated with 3-MA (autophagy inhibitor) [20]. In addition, Redondo-Castro and colleagues (2017) also observed that preconditioning human BM-MSCs with inflammatory factors (IL-1α, IL-1β, TNF-α, and INF-γ) increased the release of anti-inflammatory factors by BM-MSCs [39]. Moreover, the addition of conditioned medium from BM-MSCs preconditioned with IL-1 to an immortalized microglial cell line (BV2) stimulated with LPS reduced the release of pro-inflammatory factors and increased anti-inflammatory IL-10 secretion, promoting a less reactive state in microglial cells [39]. Another studied strategy was the pretreatment of UC-MSCs with IFN-γ. This strategy, similar to the previous, induced the production of a variety of anti-inflammatory factors [40].

##### Ultrasounds and Electromagnetic Fields

Three studies explored the effects of electromagnetic fields on BM-MSCs and have reported effects on apoptosis, increased cell viability and proliferation, and neuronal differentiation. Urnukhsaikhan and colleagues (2016) reported that treatment of BM-MSCs with electromagnetic fields increased the expression of several neural markers, promoted the activity of factors involved in neuronal differentiation and apoptosis regulation, and decreased cell death in an intensity- and time-dependent manner [41]. The authors point out that the PI3K/Akt/Bad signaling pathway might be involved in the neuroprotective effects of electromagnetic fields [41]. Moreover, BM-MSCs stimulated with low-intensity pulsed ultrasounds presented higher cell viability, migration, and neurotrophic factor expression in vitro [42]. Confirming these results, another study revealed that BM-MSCs pretreated with low-frequency pulsed electromagnetic fields proliferated faster and had greater mRNA expression of growth factors than naivenaive BM-MSCs [43].

##### Manipulation of Cell Culture Supplementation

Three studies evaluated the impact of adding various cell culture supplements on the secretome, proteome, and profile of the MSCs. Yamauchi and colleagues (2014) concluded that BM-MSCs cultured with platelet lysate (PL) and granulocyte-colony stimulating factor (G-CSF) presented higher proliferation when compared with BM-MSCs cultured in fetal calf serum or PL alone [25], suggesting that this strategy could be used for MSCs expansion. In MSCs derived from AD, the CM recovered from AD-MSCs supplemented with basic fibroblast growth factor (bFGF), and B27 triggered a significant increase in metabolic viability and cell survival in vitro [27]. Additionally, AD-MSCs cultured in a medium containing neuregulin1-beta1, basic fibroblast growth factor, platelet-derived growth factor and forskolin, presented increased secretion of brain-derived neurotrophic factor (BDNF), glial cell-derived neurotrophic factor (GDNF), VEGF-A, and angiopoietin-1 [26], supporting that this type of strategy leads to enhanced secretion of growth factors that might result in improved functional improvement.

##### Exposure to Lesion Mediators

The pretreatment of MSCs with lesion mediators, such as brain tissue extracts from animal models of disease, conditioned media from lesioned cells, or even plasma from patients, has been studied as a strategy to prime MSCs to the lesion microenvironment to stimulate their therapeutic response. The incubation of BM-MSCs with brain tissue extract from the traumatic brain injury (TBI) rat model increased the secretion of several growth factors (BDNF, NGF, VEGF, and HGF) in a time-dependent manner [35]. Another approach demonstrated that BM-MSCs’ incubation with serum from stroke patients decreased the number of senescent BM-MSCs and increased their proliferation [36]. Additionally, proteins related to the activity of chemokines, growth factors, cytokines, cell adhesion molecules, extracellular matrix proteins, serine/threonine kinases, and metallopeptidases showed differential expression between BM-MSCs treated with serum from stroke patients and BM-MSCs treated with serum from controls [36].

In another context, the incubation of BM-MSCs with bFGF, endothelial growth factor (EGF), and spinal cord injury (SCI) patient’s plasma (referred to as NRLM) increased BM-MSCs proliferation regardless of the donor’s age [37]. These cells also presented increased adipose differentiation potential. The secretome of NRLM-MSCs had increased levels of adiponectin, angiogenin, HGF, and other growth factors [37]. Using another approach, Lv and colleagues (2016) reported that BM-MSCs incubated with CM from in vitro cultures of OGD-activated microglia increased levels of GDNF secretion [38]. Overall, the results from these studies show that the exposure to the lesion microenvironment modulates the response of MSCs, namely by increasing their proliferation and boosting the secretion of factors important for neural cells.

#### 3.1.2. Low Oxygen Pressure

Typically, MSCs are cultured in an atmospheric oxygen level (20–21%, i.e., normoxia). Nonetheless, one of the most researched strategies to prime MSCs and enhance their survival in the lesion microenvironment is the manipulation of oxygen pressure during a determined period. The definition of the oxygen pressure that constitutes hypoxia varies between studies, with the majority considering hypoxia when O_2_ pressure varies between 0.1 and 5%. Another protocol that is emerging is the use of physioxia, i.e., in vivo physiological oxygen levels found in the niche from where the MSCs were isolated. Only one of the ten studies included in this section used physioxia as a preconditioning method, while all the other studies used HP. Most of the studies carried out preconditioning for 24 h; however, four of them studied the effect of using different preconditioning times.

UC-MSCs treated with physiological oxygen levels had increased proliferation and survival [24]. Nevertheless, this effect on MSC proliferation and survival has been reported after both physioxia (5% O_2_) and HP. One study reported that when BM-MSCs were subjected to preconditioning by exposure to 1% O_2_ for 6, 12, or 24 h, there was an increase in BM-MSCs proliferation, especially for the 12 h of preconditioning [34]. Exposure to HP increased the expression or secretion of numerous proteins, such as HIF-1α, erythropoietin (EPO), and trophic factors, as well as the expression of pro-survival genes [9,29,30,33]. The duration of HP appears to be critical. Analysis of the effects of the duration of the HP exposure (4–48 h) showed a peak for the effects induced by HP at 8 h. Additionally, HP for 12 h led to increased labeling for MAP-2, neurofilament light chain [34], and NeuN [31], suggesting that this strategy could promote MSCs differentiation towards neuronal cells. HP also contributed for the increased levels of proteins linked to cell migration [34].

Moreover, the combined action of HP and the addition of G-CSF to the cell culture medium boosted the protective effects of HP [34]. Moreover, UC-MSCs exposed to 1% O_2_ had increased VEGF levels, suggesting that HP influenced the pro-angiogenic effect of these cells [33]. HP was also reported to increase the expression of the stromal cell-derived factor-1 and levels of EPO receptor, both pro-angiogenic factors [31]. Regarding the impact of HP duration, BM-MSCs exposed to 0.5% O_2_ for 24, 48, or 72 h resulted in increased levels of GDNF, BDNF, VEGF, HIF-1α, and angiotensin-1 at shorter preconditioning times [31], which is in agreement with what was observed in the other studies [9,29]. In addition to the effects mentioned, it was also reported that exposure to 1% O_2_ intensified the paracrine effects of MSCs, even when they were under conditions of oxidative stress [32].

Concerning the effects of the HP-MSCs on other brain cells, such as microglia, it was reported that, when compared with CM from MSCs cultured under normoxic conditions, CM derived from BM-MSCs preconditioned with 1% O_2_ for 24 h decreased the apoptosis of BV2 microglia, the production of ROS and mitochondrial damage, and inhibited the secretion of pro-inflammatory microglia-associated molecules. The same study reported that HP increased the secretion of exosomes by BM-MSCs [28].

While different studies have employed variable percentages of O_2_ and preconditioning durations, the predominant approach has been the use of 1% O_2_ for 24-h. These reports show that positive outcomes of HP were consistently observed, such as enhanced survival and proliferation of MSCs, as well as an increased release of neuroprotective factors.

#### 3.1.3. Culture Scaffolds/3D Culture/Biomechanical Forces

Tissue engineering led to the development of scaffolds and 3D cultures, and these are now widely used to culture MSCs. Three studies evaluated the use of different systems to carry out preconditioning, microfluidic devices [10], encapsulation of MSCs in hydrogel scaffolds [24], and graphene oxide-substrate [23]).

Lech and colleagues (2020) studied the encapsulation of human UC-MSCs in hydrogel scaffolds made from human fibrin or human PL cultured in an environment of 5% or 21% oxygen [24]. UC-MSCs cultured in PL and human fibrin scaffolds exhibited elevated expression of Nestin, β-Tubulin III, Neurofilament 200, and Glial Fibrillary Acidic Protein (GFAP) in both oxygen concentrations (21% and 5%), which indicates increased differentiation of MSCs towards cells of neural lineage [24]. Furthermore, when cultured in 5% oxygen, the levels of GDNF, BDNF, EGF, IL-1β, and IL-6 increased while the levels of VEGF-A, bFGF, and TGF-β1 decreased [24]. The increased release of growth factors (NGF and GDNF) also occurred with the use of graphene oxide scaffolds, supporting AD-MSCs attachment and regulating cell adhesion and function [23].

Biomechanical forces present in the stem cell niche are crucial for the definition of fundamental stem cell properties, such as self-renewal, motility, homing, and determination of cell fate [45]. For instance, wall shear stress is a biomechanical stimulus that aims to replicate the fluid frictional forces present on the vascular lumen. These forces can stimulate MSCs to produce several anti-inflammatory factors. A study suggests that this stimulus regulated pathways involved in the modulation of immune cell phenotypes by MSCs [10].

### 3.2. Effect of the Preconditioning Method on the Therapeutic Potential of MSCs to Treat Ischemic Brain Conditions

**Figure 3:**
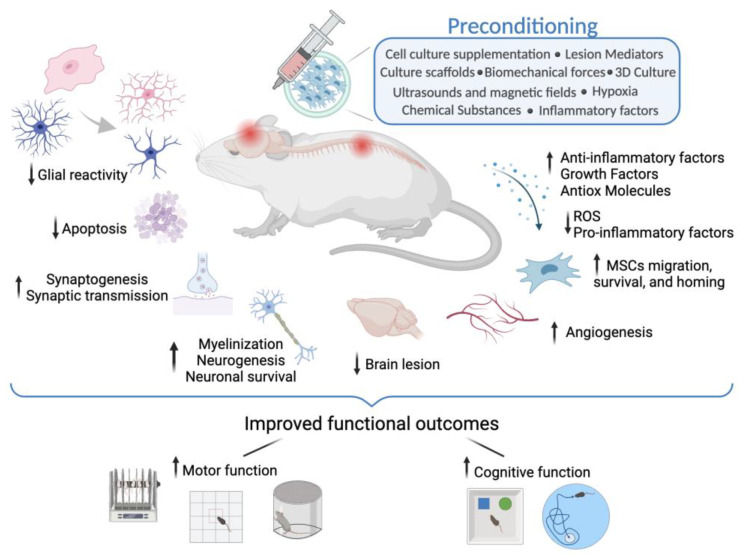
The diverse mechanisms underlying the therapeutic effects of preconditioned MSCs following brain lesions or spinal cord injuries. It includes the attenuation of glial reactivity and apoptosis, the facilitation of synaptic connectivity, and the enhancement of neuronal survival, neurogenesis, and myelinization. Additionally, it improves the recruitment and survival of MSCs at the lesion site, as well as angiogenesis and neurovascular repair, and modulates inflammatory and growth factor levels and their secretion to create a favorable microenvironment for tissue healing. Together, these processes contribute to the reduction in brain lesions and spinal cord injury severity, which translate into improved functional outcomes and neurological recovery. Abbreviations: Antiox—Antioxidant; ROS—reactive oxygen species↓—decrease; ↑—increase.

This section includes fifteen original articles that evaluated the impact of different preconditioning methods on the therapeutic potential of MSCs administered in models of ischemic brain lesions. Seven of the fifteen studies were performed in vivo and eight were developed both in vivo and in vitro. Within these fifteen articles, six different types of preconditioning were evaluated, namely the use of chemical substances and growth factors, culture supplementations, variable oxygen pressure, and exposure to lesion mediators and inflammatory factors. The relevant information for each of the articles is summarized in Table 2 and Figure 3. To facilitate the gathering of information, preconditioning methods were grouped into three groups, manipulation of cell culture supplementation, extrinsic factors, and different oxygen pressure.

**Table 2:** Studies focused on the therapeutic potential of MSCs to treat ischemic brain conditions.

Ref.	Type of Preconditioning	Preconditioning	Pathology	Type of Study	Species	Source	Administration Time (dpi)	MSC/Secretome Dose	Duration (h)	Dose Quantity Intensity	↓ Lesion Extension (vs. Naive)	Functional Improv. (vs. Naive)
[14]	Chemical substances	Roxadustat	Ischemic stroke	in vitro and in vivo	Adult male Sprague–Dawley rats	Rat BM	1	5 × 10^5^ cells	24	10 μmol/L	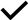	n.e.
[16]	Chemical substances	Isoflurane	Ischemic stroke	in vitro and in vivo	Rats	Rat BM	-	2 × 10^6^ cells	2, 4, 6, 12 and 24	1–10%	n.e.	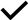
[19]	Chemical substances	Hydrogen sulfide donor	Ischemic stroke	in vitro and in vivo	Adult male Wistar rats	Rat BM	1	2 × 10^6^ cells	5, 15, 30, 60, 120 and 240 min	0.1, 0.5, 1, 5, 10 and 50 μM in vitro and 1 μM in vivo	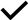	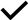
[46]	Chemical substances and growth factors	Deferoxamine	Perinatal Asphyxia	in vivo	Adult female Wistar rats	Human AD	2 h after birth and P7	16 µL of secretome (containing 6 µg of protein from 2 × 10^5^ MSCs)	48	400 µM	n.e.	n.e.
[25]	Different culture supplementation	HPL and G-CSF	Ischemic stroke	in vitro and in vivo	Adult male Sprague–Dawley rats	Human BM	7	5 × 10^5^ cells	?	5% HPL + 0.1 μM G-CSF	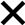	n.e.
[47]	Different oxygen pressure	Hypoxia	Ischemic stroke	in vivo	Adult male Sprague–Dawley rats	Rat BM	0.5 and every 2 days for 28 days	CM from MSC cultured in 80% confluence	12	3% O_2_	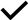	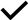
[9]	Different oxygen pressure	Hypoxia	Ischemic stroke	in vitro and in vivo	Adult male Sprague–Dawley rats	Rat BM	1	2 × 10^6^ cells	0, 4, 8, 12, and 24	1% O_2_	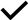	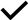
[48]	Different oxygen pressure	Hypoxia	Hemorrhagic stroke	in vivo	Adult male C57BL/6 mice	Rat BM	3 and 7	10^6^ cells	24	0.1–0.3%	n.e.	n.e.
[31]	Different oxygen pressure	Hypoxia	Ischemic stroke	in vitro and in vivo	Adult male Wistar rats	Rat BM	1	10^6^ cells	24, 48, 72	0.5% O_2_	n.e.	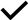
[49]	Different oxygen pressure	Hypoxia	Ischemic stroke	in vivo	Adult male C57BL/6 mice	Rat BM	3, once a day for 3 days	10^6^ cells	24	0.1–0.3%	n.e.	n.e.
[50]	Different oxygen pressure	Hypoxia	Ischemic stroke	in vivo	Adult male C57BL/6 mice	Rat BM	1	10^6^ cells	24	0.1–0.3%	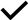	n.e.
[51]	Different oxygen pressure	Hypoxia	Neonatal stroke	in vivo	P7 male Wistar rats	Rat BM	6 h	10^6^ cells	24	0.1–0.3%	n.e.	n.e.
[36]	Exposure to lesion mediators	Stroke patient serum	Ischemic stroke	in vitro and in vivo	Adult male Sprague–Dawley rats	Human BM	1	2 × 10^6^ cells	-	10%	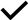	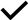
[40]	Inflammatory factors	Recombinant human IFN-γ	Periventricular leukomalacia	in vitro and in vivo	P4 Sprague Dawley rats	Human UC	0	10^6^ cells	24	10 ng/mL	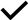	n.e.
[46]	Inflammatory factors	TNF-α+IFN-γ	Perinatal Asphyxia	in vivo	Adult female Wistar rats	Human AD	2 h after birth and P7	16 µL of secretome (containing 6 µg of protein from 2 × 10^5^ MSCs)	48	10 ng/mL TNF-α and 15 ng/mL IFN-γ	n.e.	n.e.

AD—adipose tissue; BM—bone marrow; cm—centimeter; dpi—days post-injury; G-CSF—granulocyte-colony stimulating factor; h—hours; HPL—human platelet lysate; IFN—interferon; L—liter; min—minute; n.e.—not evaluated; P—postnatal day; TNF—tumor necrosis factor; UC—umbilical cord; 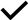
—yes; 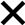
—no; ↓—decrease; ?—not reported.

#### 3.2.1. Manipulation of Cell Culture Supplementation

Although it may not be considered as preconditioning, some studies identified in the previous section highlight the potential of using different culture supplementation to influence MSCs characteristics, i.e., proliferation. Thus, it is important to assess in in vivo models of disease the impact of administering MSCs cultured under different supplemented media. Yamauchi and colleagues (2014) studied the effects of adding PL and G-CSF to the culture medium of BM-MSCs. Administration of 0.5 × 10^6^ MSCs, cultured in media supplemented with PL and G-CSF, to animals with permanent middle cerebral artery occlusion (MCAO), improved their motor performance in the rotarod test. Additionally, there was no difference between these two types of supplementation on the migration, survival, and neural differentiation of the MSCs in the infarcted brain [25].

#### 3.2.2. Extrinsic Factors

Some studies explored the impact of adding inflammatory factors to the culture medium of MSCs on the neuroprotective capacity of these cells. Two reports included in this review analyzed the effect of adding IFN-γ or TNF-α to the culture medium of MSCs to improve its potential for the treatment of two types of neonatal diseases, periventricular leukomalacia [40] and perinatal asphyxia [46]. Despite using the same type of preconditioning, the studies used different preconditioning times (24 h [16] and 48 h [46]), different sources (human UC [40] and human AD [46]) and different cell doses (0.2 × 10^6^ cells [40] and 1 × 10^6^ cells [46]). In liposaccharide (LPS)-induced neonatal periventricular leukomalacia, the CM from IFN-γ-UC-MSCs significantly improved myelination [40]. This strategy also reversed brain damage compared to UC-MSC-CM, suggesting that preconditioning UC-MSCs with IFN-γ improves their neuroprotective effects [40]. In perinatal asphyxia, the treatment with secretome from IFN-γ and TNF-α preconditioned MSCs decreased oxidative stress in the hippocampus when compared with vehicle-treated mice [46]. It was also shown that this strategy hampered neuroinflammation via a decrease in nuclear NF-κB/p65 levels and a decrease in microglial reactivity. Moreover, the administration of secretome from preconditioned cells also led to a decrease in apoptosis of hippocampal cells [46]. This strategy also improved sensorimotor function, as observed by the negative geotaxis test and the cliff aversion. Motor function was analyzed in the rotarod test, as well as anxiety and recognition memory [46]. The authors evaluated not only the effects of preconditioning with inflammatory factors but also studied a chemical agent, deferoxamine (DFX), a hypoxia-mimetic agent. DFX-preconditioning increased the production and secretion of pro-angiogenic, neuroprotective, antioxidant, and anti-inflammatory molecules by AD-MSCs [46]. Intranasal administration of the secretome from DFX-MSCs fully reversed the oxidative stress caused by the injury [46]. Similar to what happened with the inflammatory factor’s preconditioning, this strategy also decreased neuroinflammation, brain cells’ apoptosis, and led to an improvement in sensorimotor function, motor function, and memory [46]. Moreover, the MSC-secretome administration yielded similar effects whether the MSCs were preconditioned with DFX or TNF-α-+IFN-γ, improving the outcomes following perinatal asphyxia [46]. However, the authors did not evaluate the impact of administering naive cells versus the preconditioned cells, so it is not possible to assess whether preconditioning with these factors led to a potentiation of the cells’ therapeutic effect in this model.

Three other chemical agents have been studied in ischemic stroke models, with a dose range between 0.5 × 10^6^ and 2 × 10^6^ BM-MSCs. Roxadustat preconditioning increased the survival and proliferation rate of BM-MSCs after administration to a permanent MCAO model [14]. In addition, this strategy reduced the infarct volume and improved neurological recovery [14]. Furthermore, the authors report an improvement in neuronal survival and a decrease in microglial activation, along with a decrease in inflammatory cytokine levels [14].

Hydrogen sulfide, a signaling molecule in the central nervous system, can regulate ion channels, neurotransmitter functions, and other intracellular signaling molecules [52]. Accumulating evidence highlights hydrogen sulfide as a potent neuroprotective agent, demonstrating cytoprotective, anti-inflammatory, antioxidant, and anti-apoptotic effects in the central nervous system [53]. Moreover, this molecule was reported to increase neural stem cell proliferation and neuronal differentiation and revert the decrease in neurogenesis induced by hypoxic insults [54]. Preconditioning of BM-MSCs with hydrogen sulfide enhanced the therapeutic effects of these cells after permanent MCAO, reducing the infarct volume, neuronal loss, increasing vessel density, and the up-regulating BDNF and VEGF in ischemic tissue. This strategy also triggered the recovery of the neurological deficits [19].

Apart from these compounds, preconditioning of MSCs with isoflurane, a volatile anesthetic, and a known neuroprotective agent, increased the migration and engraftment of BM-MSCs in brain ischemia induced by permanent MCAO while increasing functional and neurological recovery [16].

Another approach for stimulating the MSCs consists of culturing these cells in serum from patients who have suffered an ischemic stroke. The administration of 2 × 10^6^ BM-MSCs, previously incubated with serum from stroke patients to the MCAO model, increased neurogenesis, improved mNSS, and decreased brain lesions [36].

In summary, the eight articles analyzed administered doses between 0.5 × 10^6^ and 2 × 10^6^ cells, with the higher dose being the most studied. The cells used most often were human BM, and in most cases, the effects of the therapy were observed weeks after treatment, i.e., in a chronic stage of the disease model. All included studies report that the preconditioning strategy improved outcomes in vivo. Nonetheless, the lack of experimental groups that allow the comparison between non-preconditioned and preconditioned cells does hinders further conclusions on the potentiation of the MSCs therapeutic potential in some of the studies.

#### 3.2.3. Different Oxygen Pressure

Intranasal administration of 10^6^ BM-MSCs preconditioned by hypoxia (0.1% O_2_ for 24 h) in a hemorrhagic stroke model led to the migration of these cells to perilesional regions [48]. This strategy increased the levels of neurotrophic factors in the perihematoma regions, prompting tissue regeneration [48]. Moreover, this strategy stimulated neurogenesis in the subventricular zone and migration of neuronal stem cells to the perihematoma regions [48]. In addition, the administration of HP-MSCs improved the animals’ mNSS and performance in sensorimotor and motor tests while reducing brain lesions [48]. Interestingly, the administration of the same dosage of cells in a neonatal stroke model led to similar effects, reducing infarct size and blood–brain barrier disruption, as well as promoting angiogenesis, neurogenesis, neurovascular repair [51]. The rats treated with HP-BM-MSCs showed better sensory-motor and olfactory recovery [51]. Social behavior of animals with stroke were also improved by HP-BM-MSCs [51]. Others have shown that the administration of HP-BM-MSCs to models of ischemia in the adult brain increased the migration [49] and homing [9,31,50] of these cells to the site of injury. Moreover, this strategy boosted neurogenesis and angiogenesis [49], increasing local blood flow [49]. Authors also report that animals treated with these cells had decreased neuronal apoptosis [9,47,50] and decreased infarct volume [9,47,50]. At the functional level, adhesive removal test [9,49,50], rotarod [31], balance beam test [47], open field test [47], foot-fault test [9], and the mNSS [9,47] performances highlighted the potential of HP to increase functional recovery of the lesioned animals. The administration of HP-BM-MSCs also led to a reduction in neuroinflammation, observed by a decrease in microglial reactivity and a reduction in Iba1 and CD11b-positive cells [31].

In summary, seven studies used hypoxia as a form of preconditioning, with the percentage of hypoxia varying between 0.1 and 1%. In addition, the doses of MSCs used varied between 10^6^ cells and 2 × 10^6^ cells, with five of the seven studies using 10^6^ cells. All the studies used MSCs derived from rat BM. The cells were administered between day one and 28 days after the injury. It should also be noted that all the studies used only male animals. All studies conclude that HP can increase the therapeutic potential of these cells. Despite the existing heterogeneity of the protocols, it is possible to conclude that the HP of MSCs with ≤1% O_2_ can improve their therapeutic potential in different stroke animal models.

### 3.3. Effect of the Preconditioning Method on the Therapeutic Potential of MSCs for SCI and Other Traumatic Injuries Affecting the Nervous System

Nineteen original studies assessed the effect of preconditioning strategies on the therapeutic potential of MSCs for models of traumatic injuries to the nervous system. Ten studies were exclusively in vivo investigations, eight included both in vivo and in vitro analyses, and one was performed in animal models and at a clinical level. Among these nineteen studies, seven distinct forms of preconditioning were used, including biomechanical forces, chemical substances, culture scaffolds, cell culture supplementation, different oxygen pressure, exposure to lesion mediators, and ultrasound and magnetic fields. The relevant information for each of the articles is summarized in Table 3 and Figure 3. Similarly to the previous sections, these preconditioning types were organized into three subgroups: extrinsic factors, different oxygen pressure, and culture scaffolds and biomechanical forces.

**Table 3:** Studies focused on the therapeutic potential of MSCs for traumatic injuries affecting the nervous system.

Ref.	Type of Preconditioning	Preconditioning	Pathology	Type of Study	Species	Source	Administration Time (dpi)	MSC/Secretome Dose	Duration (h)	Dose Quantity Intensity	↓ Lesion Extension (vs. Naive)	Functional Improv. (vs. Naive)
[10]	Biomecha-nical forces	Microfluidic devices	Traumatic Brain Injury	in vitro and in vivo	Adult male Sprague–Dawley rats	Human BM	1 or 3	10^7^ cells/kg	3, 6 or 8	15 dyne/cm^2^	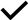	n.e.
[55]	Chemical substances	All-Trans Retinoic Acid	Spinal Cord Injury	in vivo	Adult male Wistar rats	Mouse BM	1	3 × 10^5^ cells	24	1 µM ATRA	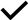	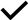
[13]	Chemical substances	Calpain inhibitor (MDL28170) and hypoxia cm or tunicamycin	Spinal Cord Injury	in vitro and in vivo	Adult male Sprague–Dawley rats	Rat BM	7	10^6^ cells	1 MDL28170 and 24 hypoxia- CM or tunicamycin	1–10 µM MDL28170 and hypoxia cm (0%) or 0, 1, 3, 10 mg/mL tunicamycin	n.e.	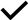
[15]	Chemical substances	Fasudil	Spinal Cord Injury	in vitro and in vivo	Adult female Sprague Dawley rats	Rat BM	7	-	12, 24, 36, 48, and 72	3, 10, 30, and 100 μmol/L	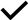	n.e.
[56]	Chemical substances	Melatonin	Spinal Cord Injury	in vivo	Adult male Sprague–Dawley rats	Mouse AD	7	Not specified	24	5 μM	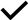	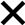
[57]	Chemical substances	Calpain inhibitor	Traumatic Brain Injury	in vivo	Adult male Sprague–Dawley rats	Rat BM	1	10^5^ cells	-	1.0 μL of 50 mM	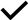	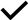
[58]	Chemical substances	Valproic acid and AMD3100	Spinal Cord Injury	in vivo	Adult male Sprague–Dawley rats	Human BM	7	10^6^ cells	3 for valproic acid and 6 for AMD3100	2.5 mmol/L of valproic acid and 20 umol/L of AMD3100	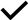	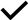
[59]	Culture scaffolds/3D culture	3D-printed collagen/silk fibroin/secretome derived from bFGF-pretreated MSCs	Traumatic Brain Injury	in vivo	Dogs	Human UC	0	Not specified	bFGF 24	N/A	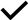	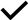
[60]	Culture scaffolds/3D culture	Collagen scaffold	Spinal Cord Injury	in vivo and clinical trial	Adult female Sprague–Dawley rats, female beagle canines aged 1 year old, and forty patients	Human UC	0	10^6^ cells in rats, 10^7^ cells in beagles, and 4 × 10^7^ cells in humans	7 days	-	n.e.	n.e.
[26]	Different culture supplementation	Growth medium with neuregulin1-beta1, bFGF, PDGF-AA, and forskolin	Peripheral nerve injury	in vitro and in vivo	Adult female Sprague Dawley rats	Human AD	0	2 × 10^6^ cells	2 weeks	200 ng/mL neuregulin1-beta1, 10 ng/mL bFGF, 5 ng/mL PDGF-AA, and 14 mM forskolin	no	n.e.
[61]	Different oxygen pressure	Hypoxia	Traumatic Brain Injury	in vivo	Adult male C57BL/6 mice	Mouse BM	1	2 × 10^6^ cells	8	1% O_2_	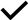	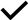
[32]	Different oxygen pressure	Hypoxia	Spinal Cord Injury	in vitro and in vivo	Adult male Sprague–Dawley rats	Rat BM	0	2 × 10^6^ cells	48 or 72	1% O_2_	n.e.	n.e.
[62]	Different oxygen pressure	Hypoxia	Traumatic Brain Injury	in vivo	Adult male C57BL/6 mice	Mouse BM	0.5 for 3 days	CM from 2 × 10^6^ cells	24	0.5% O_2_	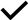	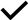
[63]	Different oxygen pressure	Hypoxia	Traumatic Brain Injury	in vivo	Adult male Sprague–Dawley rats	Human AD	7	Secretome (Not specified)	24	5% O_2_	n.e.	n.e.
[64]	Different oxygen pressure	Hypoxia	Spinal Cord Injury	in vivo	Adult male Sprague–Dawley rats	Rat BM	2 prior to ischemia/reperfusion	5 × 10^5^ cells	24	3% O_2_	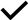	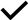
[65]	Exposure to lesion mediators	TBI tissue extracts	Traumatic Brain Injury	in vivo	Adult male Sprague–Dawley rats	Human UC	0	CM from 10^6^ cells	24	?	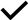	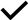
[37]	Exposure to lesion mediators	SCI patient plasma	Spinal Cord Injury	in vitro and in vivo	Adult female Sprague Dawley rats	Human BM	0 and once a week for 8 weeks	CM from 10^6^ cells	Duration of the culture	10%	n.e.	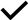
[42]	Ultrasound and magnetic fields	Low-intensity pulsed ultrasound	Spinal Cord Injury	in vitro and in vivo	Adult female Wistar rats	Rat BM	7	5 × 10^5^ cells	72	10, 30, 50, 70 mW/cm^2^, 3 min/d	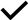	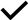
[43]	Ultrasound and magnetic fields	Low-frequency pulsed electromagnetic field	Crush-injured nerve	in vitro and in vivo	Adult male Sprague–Dawley rats	Rat BM	0	10^6^ cells	1	50 Hz, 1 mT	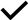	n.e.

AD—adipose tissue; ATRA—all trans retinoic acid; BM—bone marrow; cm—centimeter; dpi—days post injury; FGF—fibroblast growth factor; G-CSF—granulocyte-colony stimulating factor; h—hours; HPL—human platelet lysate; Hz—hertz; IFN—interferon; kg—kilogram; L—liter; min—minute; mT—militesla; N/A—not applicable; n.e.—not evaluated; P—postnatal day; SCI—spinal cord injury; TBI—traumatic brain injury; TNF—tumor necrosis factor; UC—umbilical cord; 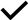
—yes; 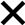
—no; ↓—decrease; ?—not reported.

#### 3.3.1. Extrinsic Factors

Several preclinical studies evaluated the impact of exposing MSCs to extrinsic factors on the potential of these cells to treat traumatic conditions affecting the nervous system.

Two studies, in models of SCI and TBI, examined the impacts of preconditioning MSCs with injury mediators. The use of the CM from BM-MSCs incubated with SCI patient’s plasma, bFGF, and EGF decreased LPS-induced nitrite release from microglia and induced neuronal regeneration more effectively in spinal cord neuronal cultures when compared with the CM from non-preconditioned MSCs [37]. The potential of this CM was also evaluated in vivo, and treatment with both CMs decreased spinal cord lesion area. Nonetheless, the CM from preconditioned MSCs improved hindlimb movement and behavior to a further extent in SCI female rats than CM from naive cells [37]. Moreover, a similar approach was used in the context of TBI. In this case, MSCs’ secretome administration led to cognitive and synaptic transmission improvements in rats subjected to TBI, with better results arising from the administration of the secretome from MSCs exposed to brain extracts from TBI rats [65]. The administration of the preconditioned secretome further increased cell proliferation, neural differentiation, and neurogenesis in the dentate gyrus, while reducing apoptosis.

As explored in the previous sections, calpain inhibitors can modulate MSC survival in vitro. This type of strategy increased BM-MSCs survival after transplantation into SCI rats while increasing the recovery of locomotor function in SCI rats [13], suggesting that this strategy boosted the therapeutic potential of these cells. Supporting these results, another study revealed that the administration of a lower dose of MSCs (0.1 × 10^6^) preconditioned with the same calpain inhibitor increased MSCs survival rate after transplantation to a rodent TBI model [57]. It was also reported that administration of the preconditioned cells reduced levels of inflammatory cytokines, number of microglia in the lesion proximity, and increased anti-inflammatory cytokines levels after TBI. Additionally, this strategy reduced lesion extension and improved neurological outcomes [57].

Valproic acid, known for its role as a histone deacetylase inhibitor affecting chromatin structure and gene expression [66], has been shown to enhance the migration of MSCs in vitro [67]. Moreover, the exposure of BM-MSCs to valproic acid prior to its administration in a rodent model of SCI resulted in increased MSCs migration to the spinal injury site and correlated with a stronger functional improvement [58].

All trans-retinoic acid (ATRA) is an activated vitamin A metabolite that was reported to stimulate autophagy in SCI models [55], as well as improve BM-MSCs survival in vitro [68]. The administration of ATRA-preconditioned BM-MSCs (0.3 × 10^6^) to a SCI rodent model increased autophagy, regulated signaling pathways involved in inflammation and immune processes and decreased pro-inflammatory cytokines [55]. The authors also reported improved hind limb motor activity and increased neuronal survival after administration of the preconditioned cells, supporting that this strategy could potentiate the beneficial effects of MSCs [55]. Fasudil, a Rho kinase inhibitor that was reported to increase MSCs migration in vitro, promoted the homing of these cells to the injury site in SCI rats [15]. The authors of the study conclude that Fasudil promotes MSCs migration both in vitro and in vivo, possibly by inducing actin stress fiber formation via the MAPK signaling pathway [15]. However, the authors did not evaluated the impact of this preconditioning strategy on functional outcomes of the lesioned animals. Another preconditioning strategy that was reported to improve MSCs migration in vivo was the use of melatonin [56]. Melatonin pretreated-AD-MSCs had higher migration to lesion site in SCI rats, presented higher differentiation into neurons, astrocytes, and oligodendrocyte lineage cells than naive AD-MSCs [56]. Nonetheless, the authors report that this strategy did not potentiate the functional improvements already observed after administration of naive MSCs to SCI rats [56]. Thus, of all the preconditioning treatments involving chemical agents, this seems to be the only strategy that was unable to promote a greater functional recovery when compared with the administration of naive MSCs.

As suggested by the in vitro studies explored in the first section, the use of magnetic and ultrasound stimulation appears to affect MSCs’ phenotype, which could translate into improved therapeutic response. Indeed, an in vivo experiment with a crush-injured nerve model revealed that, compared with injection of naive BM-MSCs, administration of 10^6^ BM-MSCs pretreated with low-frequency pulsed electromagnetic field increased myelinated axons and axonal density [43]. Nonetheless, these observations were not translated into a potentiation of sensory recoverynaive. Contrary to these findings, another study showed that transplantation of a lower dose of BM-MSCs (0.5 × 10^6^) stimulated with low-intensity pulsed ultrasound into a SCI rodent model improved locomotor function, increased BDNF and NGF levels at the injury site, and decreased cavity formation [42]. Moreover, transplantation of preconditioned cells promoted axon regeneration, suggested by increased levels of NF200 expression, and reduced gliosis, supported by decreased GFAP levels [42].

Lastly, regarding the manipulation of culture supplementation to increase MSCs therapeutic potential in vivo, Kingham and colleagues (2014) assessed the preconditioning of AD-MSCs with growth factors [26]. The authors reported that the conditioned medium of AD-MSCs stimulated neuregulin1-beta1, bFGF, PDGF-AA, and forskolin increased neurite outgrowth of cultures of dorsal root ganglia neurons as well as enhanced axon regeneration when administered to a peripheral nerve injury model, when compared to the addition of CM from non-preconditioned AD-MSCs [26]. naiveThe administration of stimulated AD-MSCs also resulted in decreased apoptosis. However, as the secretome administered included growth factors utilized for preconditioning, disentangling whether observed effects arise from the proteins secreted by stimulated cells or from the growth factors present in the secretome remains challenging. To reach conclusive results, it is imperative to collect the secretome in culture media devoid of preconditioning growth factors, achieved by washing the cells and changing the media (without the growth factors) days before collection [26].

#### 3.3.2. Differential Oxygen Pressure

The manipulation of oxygen pressure during MSC culture is the most used preconditioning technique aiming to boost MSC therapeutic potential in animal models of traumatic neurological injury. Four studies reported that the administration of HP-MSCs or their secretome decreased brain damage in TBI models [61,62,63] and improved SCI repair in rodents [64]. This approach improved cognitive and motor function in TBI models [61,62,63] as well as improved neurological scores in rodents presenting SCI [64]. Histological studies revealed that the administration of HP-MSCs or their secretome increased neurogenesis [62], promoted MSCs survival after transplantation [32], and decreased neuronal apoptosis [63], neuroinflammation [63], demyelination [61] and neurofilament impairment [61]. Additionally, levels of pro-inflammatory cytokines decreased after the administration of HP-MSCs to rats with TBI, which was accompanied by increased levels of anti-inflammatory cytokines [63]. These data support the hypothesis of increasing MSCs’ potential by using HP-MSCs or their secretome. It is crucial to emphasize the necessity of standardizing the protocols to enhance our comprehension of preconditioning effects. Among the five studies mentioned, four different O_2_ percentages (0.01, 0.03, 0.05, and 0.5) were used to condition the cells, and the HP duration ranged from 8 to 72 h. Moreover, the administered cell doses ranged from 0.5 × 10^6^ to 2 × 10^6^, with two different sources of MSCs being employed, BM and AD. Nonetheless, even though HP protocols were highly variable, positive effects of this strategy were observed across all studies.

#### 3.3.3. Culture Scaffolds and Biomechanical Forces

Culture scaffolds or biomechanical forces are being studied as strategies to stimulate mechanotransduction in MSCs. This strategy is known to induce proteomic changes in the MSCs [7], which can reflect their therapeutic potential. Diaz and colleagues (2017) applied wall shear stress, a stimulus that aims to mimic fluid frictional forces present on the vascular lumen, to human BM-MSCs [10]. The intravenous administration of MSCs subjected to wall shear stress decreased apoptotic and M1-type activated microglia in the hippocampus of TBI rats, suggesting that neuroinflammation induced by brain injury was ameliorated with this approach [10]. Nonetheless, the authors report that both the administration of naive MSCs and preconditioned MSCs resulted in a similar reduction in blood–brain barrier leakage and brain damage extension [10].

The secretome derived from UC-MSCs cultured in 3D-bFGF scaffolds was also tested as a strategy to improve recovery after TBI in dogs [59]. The purpose behind the use of these scaffolds was to increase the quality and quantity of the cells produced. Moreover, the secretome was encapsulated into 3D-printed collagen/silk fibroin scaffolds to promote its stability and retention at the injury site. This strategy ameliorated functional scores after TBI, reduced the cavity area and glial scar, and enhanced the formation of nerve fibers, myelin sheaths, and neurons in the TBI injury area [59]. Increased neurogenesis and synapse formation, angiogenesis, as well as reduced cell apoptosis, and modulate inflammatory cytokines levels were reported [59]. Although the experimental design used in this study did not allow us to distinguish what impacted more the positive results reported, whether it was the use of the secretome from UC-MSCs cultured in 3D-bFGF scaffolds or its encapsulation, these strategies appear to have an important impact on the recovery after TBI in dogs.

In summary, all strategies assessed by the three studies included in this section demonstrated positive impacts, even if the doses of the MSCs, the administration time, the duration of the preconditioning, and the source of the MSCs differ between studies.

## 4. Conclusions

Multiple studies are now focusing on how to potentiate the positive outcomes obtained after the administration of MSCs into several models of disease affecting the central nervous system. One of the approaches is the preconditioning of the cells prior to administration, meaning manipulating the microenvironment of MSCs in vitro. This systematic review explored studies that used different strategies to precondition these cells. Overall, these studies support the use of these approaches for improved functional outcomes, as well as decreased lesion extension. The most used preconditioning strategy was HP, with most reports using O_2_ pressure levels below 1% to precondition the MSCs for at least 24 h.

Preconditioning strategies explored in this systematic review have shown promising potential to enhance the therapeutic capabilities of MSCs via various pathways. These pathways include the reduction in oxidative stress, decreased apoptosis, and regulation of immune cell phenotypes. MSCs specifically reduce oxidative stress by decreasing ROS production and lipid peroxidation, acting through antioxidant enzymes. Additionally, MSCs have an anti-apoptotic effect by activating cell survival pathways and modulating cell cycles, increasing cell proliferation and migration capacity, attenuating senescence, enhancing cell adhesion, and releasing extracellular matrix modulators. The regulation of cellular phenotypes by MSCs has been achieved through the upregulation of growth factors and anti-inflammatory cytokines. Additionally, in lesion models, the administration of preconditioned MSCs triggers a cascade of pathways that contribute to the reported improved functional outcomes and enhanced tissue repair. These pathways include not only the reduction in apoptosis and the regulation of cellular phenotypes but also the enhanced migration and survival capacity of MSCs, as well as the promotion of myelination and angiogenesis.

Among the articles included, few studied the combination of different preconditioning strategies. This approach could trigger synergistic effects, thus having the potential to heighten the therapeutic benefits observed from a single preconditioning method.

In future studies, it would be crucial to understand whether the use of different MSC sources could impact the potential of preconditioning these cells. It has been observed that certain sources yield MSCs with a higher proliferative capacity and other enhanced characteristics. However, based on the studies included in this review, it is not possible to directly conclude if preconditioning strategies could be more beneficial to MSCs from some sources than others. Nonetheless, since similar strategies are applied to different sources for preconditioning, it can be suggested that all sources would likely benefit from preconditioning.

Another point for future studies would be to assess if the limitations of MSC therapy could be overcome by using preconditioning strategies. For instance, the donor variability found in MSCs, which is now starting to be reported, could potentially be normalized by using preconditioning strategies, thus uniformizing the potential of MSCs from different donors. In a similar fashion, it is imperative to acknowledge the challenges posed by certain types of preconditioning, particularly those involving chemical agents, due to the lack of knowledge regarding their safety implications for in vivo usage. Additionally, since some of the studies show that preconditioning the cells enhances their survival and proliferation in vitro, this strategy could also be used to expand the MSCs more easily and, therefore, obtain a higher yield, which will be crucial for the jump of MSCs towards clinical applications.

A crucial aspect to increase the translational potential of the generated knowledge is the inclusion of a comparison group administered with naive MSCs/secretomes to elucidate if the preconditioning strategy could, in fact, enhance the potential of MSCs. Moreover, there are several strategies that showed great promise in boosting MSC effects in vivo, and it is important to standardize these approaches, namely the extent of treatment (i.e., dosage, O_2_ pressure) and its duration. Addressing these points will contribute to a quicker translation to clinical applications because it would allow a more effective comparison between studies. Moreover, most of the studies administered a high number of MSCs (around 0.1 to 1 × 10^6^), which can compromise the conclusions, i.e., the benefits observed with preconditioning could be due to the high number of MSCs used.

## Supplementary Materials

The following are available online at https://www.mdpi.com/article/10.3390/cells13100845/s1, Table S1: PRISMA 2020 checklist. Reference [69] is cited in the supplementary materials.

## Abbreviations

3-MA	3-Methyladenine
AD	adipose tissue
ATRA	all trans retinoic acid
BDNF	brain-derived neurotrophic factor
bFGF	basic fibroblast growth factor
BM	bone marrow
CM	conditioned medium
COX2	cyclooxygenase-2
DFX	deferoxamine
DFX-MSCs	MSCs incubated with DFX
EGF	endothelial growth factor
EPO	erythropoietin
G-CSF	granulocyte-colony stimulating factor
GDNF	glial cell-derived neurotrophic factor
GFAP	Glial Fibrillary Acidic Protein
HIF	hypoxia-inducible factor
HP	hypoxia preconditioning
HP-MSCs	MSCs subjected to HP
Iba1	ionized calcium binding adaptor molecule 1
IFN	interferon
IFN-γ-UC-MSCs	UC-MSCs stimulated with IFN-γ
IL	interleukin
LDH	lactate dehydrogenase
LPS	liposaccharide
MAP-2	microtubule-associated protein 2
MCAO	middle cerebral artery occlusion
mNSS	modified neurological severity score
MSCs	mesenchymal stem cells
NGF	neural growth factor
NRF2	Nuclear Erythroid 2-Related Factor 2
NRLM-MSCs	MSCs incubated with bFGF, EGF, plasma from SCI patients
OGD	oxygen and glucose deprivation
PD	placenta-derived
PI3K	phosphoinositide-3 kinase
PL	platelet lysate
ROS	reactive oxygen species
SCI	spinal cord injury
TBI	traumatic brain injury
TGF	transforming growth factor
TNF	tumor necrosis factor
UC	umbilical cord
UC-MSCs-CM	CM from naive UC-MSCs
VEGF	vascular endothelial growth factor
